# Spacetop: A multimodal fMRI dataset unifying naturalistic processes with a rich array of experimental tasks

**DOI:** 10.1038/s41597-025-05154-x

**Published:** 2025-08-22

**Authors:** Heejung Jung, Maryam Amini, Bethany J. Hunt, Eilis I. Murphy, Patrick Sadil, Yaroslav O. Halchenko, Bogdan Petre, Zizhuang Miao, Philip A. Kragel, Xiaochun Han, Mickela O. Heilicher, Michael Sun, Owen G. Collins, Martin A. Lindquist, Tor D. Wager

**Affiliations:** 1 Dartmouth College, Hanover, NH USA; 2 Johns Hopkins University, Baltimore, MD USA; 3 Emory University, Atlanta, GA USA; 4 Beijing Normal University, Beijing, China; 5 University of Wisconsin-Madison, Madison, WI USA; 6 University of California, Irvine, CA USA

**Keywords:** Perception, Language, Decision

## Abstract

Cognitive neuroscience has advanced significantly due to the availability of openly shared datasets. Large sample sizes, large amounts of data per person, and diversity in tasks and data types are all desirable, but are difficult to achieve in a single dataset. Here, we present an open dataset with N = 101 participants and 6 hours of scanning per participant, including 6 multifaceted functional tasks, 2 hours of naturalistic movie viewing, structural T1 images and multi-shell diffusion imaging as well as autonomic physiological data. This dataset’s combination of sample size, extensive data per participant (>600 iso-hours of data), and a wide range of experimental conditions — including cognitive, affective, social, and somatic/interoceptive tasks — positions it uniquely for probing important questions in cognitive neuroscience.

## Background & Summary

Neuroimaging has significantly advanced our understanding of the dynamics of the human mind, specifically at the macroscale level. This progress – which nicely complements human behavioral studies and animal models of micro-meso scale neuroscience – is driven by developments in analytic methods, the high quality of imaging data, the increase in practices of sharing open datasets, and extensive data collection efforts. In large scale studies, two primary approaches are employed: either sampling a large number of participants to enhance statistical power for group average analyses (increasing between-subject sampling) or collecting extended hours within participants to better detect specific processes (increasing within-subject sampling)^1^. Here, leveraging both between-subject and within-subject sampling, we introduce “Spacetop” – a dataset that adeptly balances a large sample size of 101 participants with 6 hours worth of neuroimaging data per participant. Originally developed for functional alignment of individuals via naturalistic movie data and modeling of topological brain spaces across diverse cognitive tasks, this dataset is positioned to deepen our understanding of individual brain function, and also highlight individual differences in cognitive processes, given the statistical power that this dataset offers.

Another line of consideration in data collection has been the choice between naturalistic and experimental paradigms. Recently, naturalistic stimuli have proven invaluable for scientific advances in the neuroimaging community^2–4^, providing rich contexts for exploring a wide range of features from perceptional, situational, to abstract conceptual levels. They offer ecological validity, enabling deeper insights into sensory perception or abstract processing, as the meaning of a stimulus unfolds in a dynamic context in the real world. For example, research in behavioral neuroscience reveals that even robust findings in the primary visual cortex (V1) demonstrate different tuning profiles towards naturalistic movie stimuli and non-naturalistic Gabor filters^5^. This suggests that the neural mechanisms of natural vision may differ from our scientific understanding of vision, due to the large dependence on highly controlled experiments. To further illustrate this notion, studies using naturalistic speech narratives uncover distributed representations of semantic knowledge, contrary to past findings of lateralization in semantic knowledge^6^. However, it is important to note the inherent challenges of these naturalistic approaches, as they often yield noisier parameters and require complex modeling approaches compared to traditional experimental designs.

For these reasons, experimental designs are indeed crucial to understanding human brain function. In fact, causal relationships of treatment effects are only guaranteed through manipulation and randomization of variables^7–10^. A well-defined parameter space in experiments allows for testing specific hypotheses, developing precise models, and increasing efficiency and statistical power. The rigor and precision of experimental designs are precisely why they are considered the gold standard in biomedical science and why regulatory agencies require randomized controlled trials for drug approvals. These designs effectively isolate the variables of interest and minimize the influence of confound variables. As is well known, fMRI data, specifically the Blood Oxygen Level-Dependent (BOLD) response, is delayed and cumulative in nature compared to neural activity, thereby complicating signal attribution to a variable of interest. However, extensive research on the signal profile in tandem with optimized experimental designs^11–13^ have mitigated these limitations, enhancing the accuracy of interpretations of the defined parameter space, driving advancements in human neuroimaging for decades.

By integrating both naturalistic and experimental stimuli, we are able to uncover common neural properties that bridge the gap between ecologically valid contexts and experimentally controlled conditions. This integrated approach in past studies has led to significant insights. For example, in an fMRI study on action perception, researchers find several visual processing regions that are consistently activated for both static and dynamic conditions, indicating invariance to form or presentation modality^14^. Similarly, a study on numerical processing using electrocorticography^15^ revealed that the intraparietal sulcus, active in numeracy tasks in experimental settings, are also active during social conversations in naturalistic settings involving numerical concepts. This combined approach not only identifies the homology between the two different settings, but also highlights the subtle distinctions inherent in each process, offering a more holistic understanding of human brain functions.

Harnessing the power of both approaches, our dataset includes 120 minutes of naturalistic movie data and audio narratives, complemented by subjective ratings from participants, and a range of experimental tasks, including somatic, social, cognitive, and affective experimental conditions. Such an approach leverages both ecological validity and statistical power. We envision this dual approach to serve as a versatile tool for probing brain function (For a comparative overview of this dataset in relation to other large scale datasets, see Table 1).

**Table 1:** Comparison of major open fMRI datasets.

Dataset	Between-/Within-Subjects			Task Type		Data Type	
	Functional Isohours	# of Subjects	Hours per Subject	# of fMRI Tasks	Naturalistic or Experimental	Somatic Pain	Physiological Data
Spacetop	606	101	6	9	Both	Yes	Yes
BioBank	0.1	500,000	0.1	2	Resting-state/Experimental	No	No
ABCD	2,000	10,000	0.5	3	Experimental	No	No
HCP	3,300	1,200	2.75	7	Experimental	No	No
SuperStruct	314	1,570	0.2	1	Resting-state	No	No
NSD	320	8	40	1	Experimental	No	No
Narratives	345	345	1	1	Naturalistic	No	No
Midnight Scan Club	110	10	11	3	Experimental	No	No
BOLD5000	96	4	24	1	Experimental	No	No

This dataset uniquely positions itself amongst a selection of notable publicly available neuroimaging datasets. There are many great open datasets, majority shared on OpenNeuro, amounting to 1,300 public datasets. A subset of the datasets are summarized here for comparison, based on key examples referenced in Naselaris and colleagues^1^, who summarized datasets based on the distribution of within/between-subjects of each dataset. Summary of nine publicly available neuroimaging datasets, comparing scale, experimental design, and inclusion of pain- or physiology-related data is listed in the table. **Functional isohours** refers to the approximate total number of functional scanning hours across subjects, i.e. multiplication of **of Subjects** and Hours per subject. **Hours per subject** indicates the average duration of fMRI data collected per participant. For further details on openly shared datasets, please see Botvinik-Nezer and Wager (2024).

One primary use case of this dataset, Spacetop, would be to develop functional alignment techniques to address the challenges of individual differences in neuroimaging. Alignment methods^16^, such as hyperalignment, connectivity alignment, and shared response models, provide solutions to narrow the gap across individuals and help uncover group-level neural processes. Since using functional training data from the same individuals is essential for implementing these techniques effectively, the 90 minutes of movie data provides an ideal test environment. Additionally, the inclusion of experimental tasks allows for rigorous test comparisons between different functional alignment methods, while allowing for an opportunity to establish convergent construct validity between experimental and naturalistic tasks.

## Methods

### Participants

**Fig. 1:**
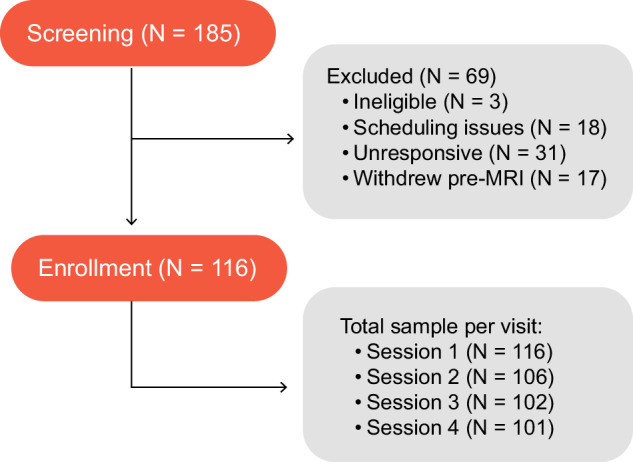
Consort diagram. A total of 185 participants enrolled in the study. Of these, 116 participants completed session 1, 106 completed session 2, and 102 completed session 3. A total of 101 participants completed all four sessions.

This dataset includes 101 adult participants (mean ± s.d. age: 24.7 ± 5.5 years; 69 males, 45 females, 2 others). Data were collected from December 2020 to July 2022 at the Dartmouth Brain Imaging Center. Participants were healthy individuals, with normal or corrected-to-normal vision and hearing, no recent psychiatric or neurological diagnoses within the past six months, no MRI contraindications, and no chronic pain. We determined eligibility via a general health questionnaire, a pain safety screening form, and an MRI safety screening form. Additionally, individuals with self-reported chronic pain who anticipated discomfort while lying in an MRI scanner were not enrolled. All participants were right handed. Participants were recruited from the area of New Hampshire and Vermont as well as the Dartmouth college student body. The institutional review board (IRB) of Dartmouth College approved to conduct the study and share the data (CPHS STUDY00031937), and all participants provided written consent. The consent form included a section on data sharing, which explicitly states that deidentified data may be shared via a publicly available data archive. In addition, participants were informed that, for all potentially shared data, every reasonable effort would be made to remove identifiers from the data that would indicate any connection to the participant. Participants reconsented for each visit. A consort diagram is included to illustrate the enrollment and study attrition rate across four sessions (Fig. 1).

### Overview of experimental procedures

**Fig. 2:**
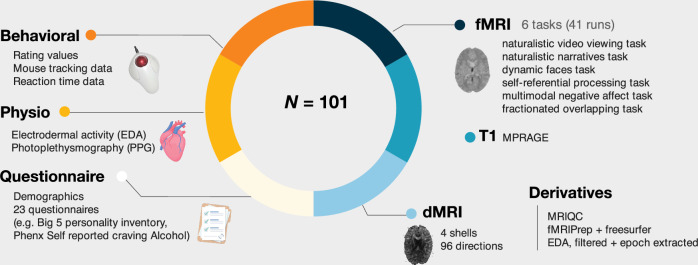
Overview of acquired data. Dataset includes N = 101 participants worth of data, including (*clockwise*): 1) functional BOLD echo-planar imaging with cognitive tasks (TR = 460 msec, MB = 8), 2) a T1-weighted anatomical scan, 3) a multi-shell diffusion weighted MRI (dMRI) scan, 4) a battery of questionnaires prior to scanning, 5) physiological data collected during scanning, and 4) behavioral data collected during scanning.

This dataset includes multimodal data from four in-person neuroimaging sessions and an at-home questionnaire survey session. Upon arrival for each in-person neuroimaging session, participants were invited to a separate behavioral testing facility to complete a behavioral informational session, where they signed consent forms and then had scripted task instructions read to them, accompanied by visual aids of upcoming tasks. Participants then completed a behavioral practice task on a computer to reinforce their understanding of the task. After the behavioral information session, participants were invited to the MRI scanning facility, in which they completed experimental tasks with concurrent fMRI and physiological recordings of skin conductance and photoplethysmography (Fig. 2; see Table 2 for imaging acquisition parameters). Participants completed at-home surveys prior to the first incoming scan session. These surveys primarily assessed general psychosocial tendencies, with the aim to link them with behavioral, physiological, and neural responses observed during the neuroimaging experiments (Table 3).

**Table 2:** Description of imaging acquisition parameters.

Features	Main Task	T1w	DWI	Distortion Correction
Resolution [mm]	2.7 (iso)	0.8 (iso)	1.7 (iso)	2.7 (iso)
# Volumes	Varies per task	1	1	2
FOV [mm]	220	256	240	220
Matrix Size	82 × 82	320 × 320	130 × 130	82 × 82
TR [s]	0.46	2	4.11	7.22
TE [ms]	27.20	2.11	88.40	73.00
Flip Angle [degree]	44^°^	8^°^	90^°^	90^°^
Slice Orientation	Transversal	Sagittal	Transversal	Transversal
Phase Encoding Direction	A >> P	A >> P	A >> P	A >> P
Number of Slices	56	224	81	56
Slice Thickness [mm]	2.7	0.8	1.7	2.7
Distance Factor [%]	0	50	0	0
Order of Slice Acquisition	Interleaved	Interleaved	Interleaved	Interleaved
iPAT mode	none	GRAPPA	none	none
Multiband Acceleration Factor	8	3	3	1
Bandwidth [Hz/px]	3048	240	1700	3048

**Table 3:** Description of questionnaires.

Name of instrument	Description	Primary source
**Online Behavioral Surveys**		
a) Demographics survey	Demographics	Internal Survey
b) Jessor Demographics	Demographics	Internal Survey
c) Multidimensional Assessment of Interoceptive Awareness (MAIA-32)	Interoception	Mehling *et al*.^57^
d) PROMIS-57 Profile v2.1	Physical function, anxiety, depression, and pain	Cella *et al*.^58^
e) Canlab Pain Survey (Pain Symptoms)	Pain disorders or sensitivities	Internal Survey
f) Life Orientation Test Revised (LOT-R)	Trait levels of optimism and pessimism	Scheier *et al*.^59^
g) Positive and Negative Affect Scale	Trait levels of positive and negative affect	Watson *et al*.^60^
h) Fear of Pain (FOP)	Fear and anxiety associated with pain	McNeil & Rainwater^61^
i) Behavioral Inhibition/Behavioral Activation (BIS/BAS)	Trait levels of approach and avoidance behavior	Carver & White^62^
j) The “Big 5” Brief Inventory Brief Version	Various personality traits	Rammstedt & John^63^
k) Ego-Resiliency Scale (ER89)	Ability to respond adaptively and resourcefully to new situations	Block & Kremen^64^
l) Adverse Childhood Experience (ACE) Questionnaire	Childhood trauma (if any)	Felitti *et al*.^65^
m) Marlow-Crowne Social Desirability Scale 13-Item Short Form	Social desirability	Crowne & Marlowe^66^
n) Interpersonal Reactivity Index (IRI)	Thoughts and feelings in a variety of situations	Davis^67^
o) PhenX Alcohol Lifetime Use	Degree of lifetime alcohol use	PhenX Toolkit^68^
p) PhenX Alcohol Age of First Use	Age of first alcohol use	”
q) PhenX Alcohol 30Day Quantity and Frequency	Quantity and frequency of alcohol use	”
r) PhenX Substances Lifetime Use	Degree of lifetime substance use	”
s) PhenX Substances Age of First Use	Age of first substance use	”
t) PhenX Substance 30 Day Frequency	Frequency of substance use	”
u) PhenX Cigarette Smoking Status Adult	Status of cigarette smoking	”
v) PhenX Tobacco Age of Initiation of Use Adolescent	Age of tobacco initiation	”
w) PhenX Tobacco 30 Day Frequency	Frequency of tobacco use	”
x) Self-Report Psychopathy Scale	Identification of psychopathic behaviors	Williams *et al*.^69^
y) Balanced Inventory of Desirable Responding Short-Form (BIDR)	Tendency to self report with positive bias and impression management	Hart *et al*.^70^
z) 20-Item Prosopagnosia Index	Face recognition ability	Shah *et al*.^71^
aa) Tendency to Conform	Social conformity	Goldsmith *et al*.^72^
bb) Therapeutic Reactance	Psychological reactance	Dowd *et al*.^73^
cc) Revised Self-Monitoring	Sensitivity to expressive behavior and ability to monitor self-representation	Lennox & Wolfe^74^
dd) Concern for Appropriateness	Tendency to conform	Lennox & Wolfe^74^
**In-person Surveys (prior to fMRI scan)**		
Pain Medication Questionnaire	Pain medication administration within the last 12 hrs prior to participation	Internal Survey
Menstruation Questionnaire	Menstrual cycle phase at the time of the participation and details about participants cycle	Internal Survey

Participants completed at-home surveys prior to initial neuroimaging sessions. **Name of instrument** column indicates the short hand title of each questionnaire. **Description** indicates a short summary of the aim of each questionnaire and **Primary source** indicates the reference for each questionnaire.

### Overview of neuroimaging modalities and tasks

**Fig. 3:**
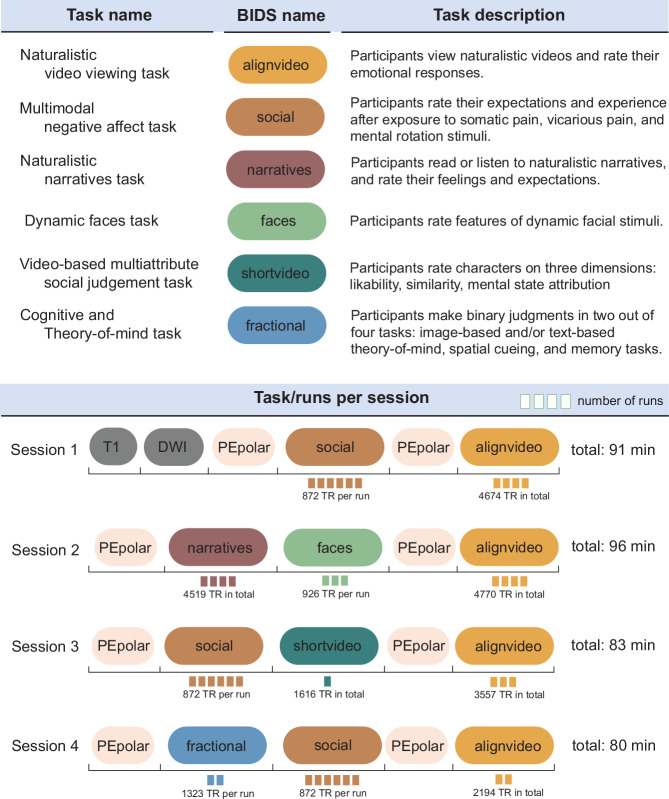
Data acquisition layout. Each neuroimaging session included task data from multiple cognitive domains. The upper panel illustrates all of the tasks included in this dataset with 1) the name of each task, 2) its corresponding Brain Imaging Data Structure (BIDS) task name, and 3) a short task description. The lower panel illustrates the task composition across four sessions. Session 1 consisted of one anatomical T1-weighted scan, multi-shell diffusion weighted image, phase-encoding polarity (PEpolar) image for distortion correction, followed by six EPI runs of the multimodal negative affect task (“task-social”), another PEpolar image for distortion correction, followed by four EPI runs of the naturalistic video viewing task (“task-alignvideo”). Session 2 started with a PEpolar image, followed by four runs of naturalistic narrative task (“task-narratives”), three EPI runs of the dynamic faces task (“task-faces”), followed by PEpolar run, and four EPI runs of the naturalistic video viewing task (“task-alignvideo”). Session 3 consisted of an PEpolar run, followed by six EPI runs of the multimodal negative affect task (“task-social”), one EPI run of the video-based multiattribute social judgment task (“task-shortvideo”), PEpolar run, and ended with three EPI runs of the naturalistic video viewing task (“task-alignvideo”). Session 4 started with an PEpolar run, followed by two EPI runs of the cognitive/theory of mind task (“task-fractional”), six EPI runs of the multimodal negative affect task (“task-social”), another PEpolar run, followed by two EPI runs of the naturalistic video viewing task (“task-alignvideo”).

**Fig. 4:**
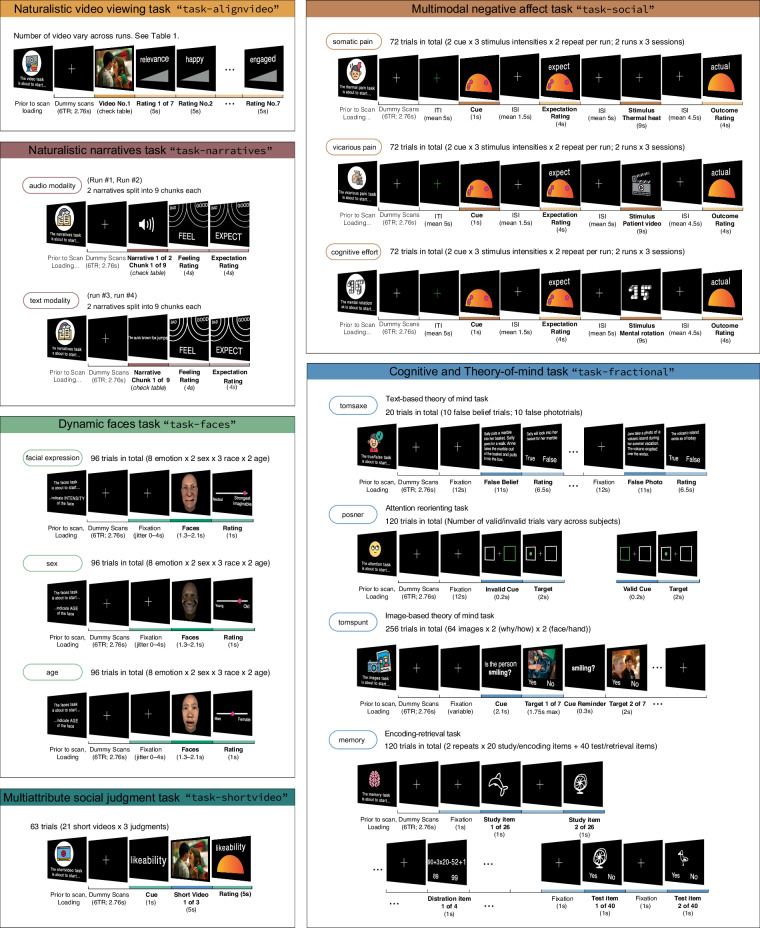
Depiction of task structure and its variations across runs. Each of the six panels refers to a task. Within each panel, “trials” are described, which are fundamental units of a run, designed to capture a specific cognitive process. Trials are repeated within runs. The number of these repetitions, which may vary across tasks, is indicated on the right side of the panel. For tasks employing a factorial design, — specifically, task-faces, task-shortvideo, task-social, task-tomsaxe, task-posner, task-tomspunt, task-memory — the experimental factors are also listed on the right. On the left side of each panel, we note any changes in rating or stimulus modality across runs. For example, task-narratives encompassed two distinct stimulus modalities: audio and text narratives, delivered in separate runs. In a similar vein, the task-social consisted of several distinct runs, each dedicated to a different domain: somatic pain, vicarious pain, and cognitive effort. Stimuli corresponding to each domain were presented exclusively within their respective runs. Lastly, task-faces included three runs, each requiring participants to judge faces based on different dimensions, such as sex and age respectively. These variations in modality and rating type across runs are indicated in bubbles on the left side of each panel.

The dataset includes three modalities of neuroimaging data: Anatomical T1-weighted image, diffusion weighted image, and functional echo-planar images (EPI) for experimental/naturalistic tasks. Each neuroimaging session encompassed a variety of tasks, each designed to probe different cognitive domains (illustrated in Fig. 3: “task description”). For example, in task-alignvideo, participants watch naturalistic videos and rate their emotional responses. Therefore, this task includes multimodal stimuli, incorporating both visual and auditory elements, and incorporates affective and social domains. Conversely, task-faces specifically focuses on face processing, involving visual recognition and interpretation of affective facial expressions. Detailed descriptions of each task are provided in subsequent subsections, denoted as “task-”. Visual representations of individual trials within each task are depicted in Fig. 4. While analysis of interest may vary across researchers, we provide a detailed overview of the key contrasts inherent to each task (Table 4). This includes a description of the primary cognitive processes, or “canonical” contrasts, which serve for understanding the core aspects of each tasks’ design and its analytical focus.

**Table 4:** Description of canonical neuroimaging contrasts per task.

Task	Contrasts	Covariates
Naturalistic video viewing task task-alignvideo	Video > baseline rating	Emotion ratings
	Encoding models of video attributes	—
Naturalistic narratives task task-narratives	Audio > Text narrative	—
	Narrative (Audio and Text) > baseline	Expectation & Feeling rating
Multimodal affective task task-social	Somatic pain > baseline	Expectation & Outcome rating
	Vicarious pain > baseline	Expectation & Outcome rating
	Cognitive discomfort > baseline	Expectation & Outcome rating
Dynamic Faces task task-faces	Age > baseline	Age rating
	Gender > baseline	Sex rating
	Facial expression > baseline	Intensity rating
Video-based multiattribute social judgement task task-shortvideo	Likeability > baseline	Likeability rating
	Similarity > baseline	Similarity rating
	Mental state attribution > baseline	Mental state attribution rating
Fractionated “Why/how” task	Why > How	Accuracy (image detection)
Fractionated “False-belief” task	False belief > False photograph	Accuracy (true/false judgment)
Fractionated “Posner” task	Invalid cue > Valid cue	Accuracy (target detection)
Fractionated “Memory” task	Encoding > baseline	Accuracy (old/new identification)
	Retrieval > baseline	Accuracy (old/new identification)

To facilitate exploration of the dataset, we highlight the primary processes associated with each task by outlining the canonical contrasts each task (listed in **Task** and **Contrasts** column). Additionally, **Covariates** indicate the participant ratings collected during each trial. These covariates could be integrated into within-subject models or included as between-subject individual difference measures after further computation. For example, the Narratives task includes contrasts such as 1) audio vs. text modality comparison, 2) narrative vs. baseline comparison, and 3) the incorporation of behavioral ratings as covariates.

### Specific tasks, stimuli and rating description

#### Generalized Labeled Magnitude Scale (gLMS)

A challenge in scientific studies of human behavior is the subjectivity of outcome measures, making ratings often non-comparable across studies. For this dataset, we used a modified semi-circular-shaped generalized labeled magnitude scale (gLMS^17^), which allows for magnitude matching across participants, originating from gustatory research and psychophysics. Two tasks – Multimodal negative affect task (“task-social”) and Video-based multiattribute social judgement task (“task-shortvideo”) – use elements of the gLMS. The scale is intended to measure subjective sensory experiences quantifiably and comparably across different sensory/cognitive domains, allowing for comparisons both between and within participants. Labels on the scale range from “No sensation” (0^∘^), “Barely detectable” (3^∘^), “Weak” (10^∘^), “Moderate” (29^∘^), “Strong” (64^∘^), “Very Strong” (98^∘^) to “Strongest sensation of any kind” (180^∘^). To mitigate scale usage bias, the scale was adapted from a vertical linear scale to a semicircular presentation, in which reported ratings are designed to be equidistant from the cursor starting point. The scale and associated tasks were presented on a desktop computer during the pre-scan behavioral instruction sessions and in-scanner fMRI tasks. The scale was adapted to each task by modifying the label of the maximum magnitude marker within the context of the task’s measures.

#### Naturalistic video viewing task 'task-alignvideo'

##### Task procedures

A naturalistic video-viewing task was administered across all four sessions. Each session consisted of multiple runs (see Fig. 3 for run composition per session); each run consisted of multiple video trials. Each trial consisted of two epochs of watching a video and rating seven emotion categories. First, participants watched a series of emotionally salient videos (“video”) and next, were intermittently prompted to provide ratings (“rating”) about how the video made them feel in relation to seven affective domains: 1) personal relevance, 2) happy, 3) sad, 4) afraid, 5) disgusted, 6) warm and tender, and 7) engaged. Participants were given 5 seconds to make each of the seven ratings; the sequence of these ratings were kept constant. A variety of 49 unique videos were presented to participants, ranging from 20 seconds to 5 min 39 seconds in duration, amounting to 86 minutes and 9 seconds across 4 sessions (See Table 5 for detailed breakdown of video duration, order and, number of TRs). Each video was played once, with no repetitions. The sequence of the videos were identical across participants, purposefully designed for functional alignment purposes.

**Table 5:** Overview of the naturalistic videos used in task-alignvideo.

Video name	Session	Run	Order	Duration (mm:ss)	TRs
idiots	ses-01	run-01	1	00:53	115
wanderers	ses-01	run-01	2	03:18	430
lioncubs	ses-01	run-01	3	01:00	130
parkour	ses-01	run-01	4	00:38	82
harrymetsally	ses-01	run-02	1	02:35	336
HB	ses-01	run-02	2	00:57	123
Islamophobia	ses-01	run-02	3	04:03	528
beach sunset	ses-01	run-02	4	00:30	65
Hugging pets	ses-01	run-03	1	01:46	230
bestfriendsweating	ses-01	run-03	2	00:59	128
cupstacking	ses-01	run-03	3	00:20	43
dancewithdeath	ses-01	run-03	4	02:16	295
beatbox	ses-01	run-04	1	02:22	308
angrygrandpa	ses-01	run-04	2	05:39	736
knifegame	ses-02	run-01	1	00:26	56
freesolo	ses-02	run-01	2	01:11	154
youth	ses-02	run-01	3	01:51	241
deer	ses-02	run-01	4	00:30	65
mediabias	ses-02	run-02	1	01:59	258
photography	ses-02	run-02	2	03:27	450
menrunning	ses-02	run-02	3	03:23	441
giving	ses-02	run-02	4	02:57	384
unefille	ses-02	run-03	1	01:56	252
captureflag	ses-02	run-03	2	02:59	389
tornado	ses-02	run-03	3	00:26	56
war	ses-02	run-03	4	01:02	134
forestfire	ses-02	run-04	1	00:47	102
iceblock	ses-02	run-04	2	00:47	102
alwaysafamily	ses-02	run-04	3	02:26	317
visswar	ses-02	run-04	4	00:33	71
mountainbike	ses-03	run-01	1	03:31	458
carflood	ses-03	run-01	2	00:20	43
fireplace	ses-03	run-01	3	00:30	65
snakes	ses-03	run-01	4	02:03	267
planetearth	ses-03	run-02	1	02:28	321
normativeprosocial3	ses-03	run-02	2	01:26	186
heartstop	ses-03	run-02	3	03:16	426
beachsunrise	ses-03	run-02	4	00:35	76
normativeprosocial2	ses-03	run-03	1	01:45	228
normativeprosocial1	ses-03	run-03	2	01:14	160
stardust	ses-03	run-03	3	03:16	426
universe	ses-04	run-01	1	00:50	108
gockskumara	ses-04	run-01	2	02:59	389
skiing	ses-04	run-01	3	00:56	121
cyclegraphics	ses-04	run-01	4	02:27	319
dogdance	ses-04	run-02	1	00:38	82
littleboat	ses-04	run-02	2	01:14	160
gangan	ses-04	run-02	3	02:18	300
tornado	ses-04	run-02	4	00:27	58

Each row represents a video that was presented during one trial. The column **Video** indicates the video filename. **Session** and **Run** indicates the session and run in which the videos were presented. **Order** indicates the order of the video played in each run. **Duration (mm:ss)** indicates the length of the video. **TRs** indicates the number of TRs, i.e. 0.46 seconds, that the video spans. Each video file follows the naming convention: {Session}_{Run}_order-{Order}_content-{Videonames}.mp4, shared in the stimuli/task-alignvideo folder.

##### Stimuli and scales

To submit affective ratings of videos, the participants were presented with a continuous, linear scale ranging from “Barely at all” to “Strongest imaginable”. Practice ratings were provided using a computer mouse, and experimental ratings were provided using an MR-compatible trackball.

#### Multimodal negative affect task 'task-social.'

##### Task procedures

Participants performed three different task domains: somatic pain (“pain”), vicarious pain (“vicarious”), and mental rotation (“cognitive”). Each task was designed as a 2 cue (high/low) x 3 stimulus intensity (high/med/low) factorial design. The three tasks were conducted repeatedly on average, one week apart across three sessions (ses-01, ses-03, ses-04). Each trial consisted of four events: first, participants passively viewed a presentation of a high or low social cue, consisting of data points that participants believed indicated other people’s ratings for the upcoming stimulus. Cues were presented for 1 second on screen (“cue”); second, participants provided ratings of their expectations on the upcoming stimulus intensity on a gLMS scale for a total duration of 4 seconds overlaid with the cue image (“expectancy rating”); third, participants passively received/viewed experimentally delivered stimuli for each of the mental rotation, vicarious pain, and somatic pain tasks for 5 seconds each (“stimulus”); lastly, participants provided ratings on their subjective experience of cognitive effort, vicarious pain, or somatic pain for 4 seconds (“outcome rating”). In total, each run was designed to last 6 minutes and 41 seconds, i.e., 872 TRs. The task was administered in ses-01, ses-03, and ses-04.

##### Stimuli and scales

Stimuli for the somatic pain task were thermal heat stimuli, administered using a TSA2 system (Medoc) with a 16-mm Peltier contact thermode, delivered to the glaborous skin of the ventral surface of the left forearm. Stimuli at three stimulus intensity levels were delivered (low: 48^°^C; medium: 49^°^C; high: 50^°^C), for a total duration of 9 seconds with a 5 second plateau. Baseline temperature was 32^°^C. To account for the delay in reaching the intended temperature, an additional two seconds were padded to both the ramp-up and ramp-down phases. As a result, the pain stimulus was 9 seconds long, with 5 seconds of peak intended temperature. To maintain consistency across conditions, the vicarious and cognitive stimuli were also padded with fixation crosses to match the stimulus duration across conditions. Stimuli for the vicarious task were videos of patients in pain, selected from the UNBC-McMaster shoulder pain expression archive database 10.1109/FG.2011.5771462^18^ and categorized into three stimulus intensity levels using the pre-normed ratings (self-reported pain rating and observer estimated-pain rating) provided from the dataset. Stimuli for the mental rotation task were images from the Ganis & Kievet 10.5334/jopd.ai; https://figshare.com/articles/dataset/A_new_set_of_three_dimensional_stimuli_for_investigating_mental_rotation_processes/1045385^19^ dataset, selecting images that were rotated 50, 100, 150 ^∘^ to account for three intensity levels.

#### Naturalistic narratives task 'task-narratives'

##### Task procedures

Participants were instructed to read or listen to 8 different narratives while in the scanner, with each narrative chunked into 9 clips. Each trial started with a fixation period with jittered durations between 2 and 8 seconds (“fixation”). During the narrative epoch, audio or text clips were presented (“audio” or “text”). Participants were then prompted to rate how they felt about the narrative (“feeling”) and what their expectations were for the upcoming narrative on how good or bad the future storyline would be (“expectation”). The entire task consisted of four runs, with two narratives, i.e. 18 narrative clips in each run. In total, each run was designed to last 7 minutes 24 seconds to 9 minutes 57 seconds, i.e., 967, 1098, 1298, 1156 TRs per run. The task was administered in ses-02.

##### Stimuli and scales

The narrative contents were manipulated across three distinct timescales: individual situations, the context of those situations, and the overarching full narrative. Each narrative includes a single main character who moves between three different contexts and, in each context, encounters three different situations (characterized by interpersonal relationships and actions). We use Polti’s 36 situations to construct these different situations^20^. The duration of the eight narratives range from 1 minute 36 seconds to 3 minutes 29 seconds (Table 6). The rating scale had two poles on the scale, each labeled “good” and “bad”, presented at the two upper corners of the screen. The center of the poles represent higher intensity; participants would report how intensely good or bad they felt about the current narrative and expected what the next narrative would be like. The scale allows for encoding two dimensions of a participants subjective experience – intensity and valence – in comparison to a linear scale.

**Table 6:** Overview of the naturalistic narratives used in the Narratives task.

Run #	Story #	Modality	Duration (mm:ss)	TRs	Words
run-01	7	audio	02:01	263	429
run-01	8	audio	01:36	209	314
run-02	5	audio	02:30	326	511
run-02	6	audio	02:04	269	447
run-03	3	text	03:11	415	573
run-03	4	text	03:29	454	626
run-04	1	text	02:43	355	490
run-04	2	text	02:40	348	480

The narrative task was operated within a single session, ses-02. There were four runs in total, with eight different narratives. Each row represents a narrative, presented within each run. The **Run** column indicates the run order in which the narrative was presented. Story represents the narrative index. **Modality** indicates the form in which each narrative was presented. **Duration (mm:ss)** indicates the length of the narrative. **TRs** indicates the number of TRs, i.e. 0.46 seconds, that the audio or text-based narrative spans. Each narrative was further divided into nine chunks labeled by situations; each chunk was presented per trial.

#### Dynamic faces task 'task-faces'

##### Task procedures

Participants were presented with 288 dynamic faces of varying race, age, sex, and facial expressions. The faces task consisted of three runs; in each run, participants were prompted to rate specific dimensions of the face: age, sex, or intensity of the facial expression. The order of the runs were pseudo-randomized based on odd and even numbered participant IDs. Participants were made aware of the rating dimension before the start of each run. In each trial, a brief video featuring a single face, displaying an expression, was played (“faces”), and participants were given 1.875 seconds to rate the face on the corresponding rating: the intensity of the facial expressions, the sex of the face, or the age of the face (“rating”). Participants indicated their responses by either clicking the mouse to lock in their rating or by allowing the cursor position at the end of the response period to serve as their recorded rating. After each rating, a fixation cross was displayed with a jittered duration of 0-4 seconds. Each of the three runs lasted 7 minutes and 7 seconds, (i.e., 914 TRs) and featured 96 facial stimuli. The task was administered within a single session, during ses-02.

##### Stimuli and scales

The stimuli were animated clips of human faces, ranging from 1.3 to 2.1 seconds, varying across four underlying factors: age, sex, race, and facial expression. The faces were either young or old (“age”), male or female (“sex”), Eastern Asian, Western Caucasian, or African (“race”) and exhibited one of eight emotions: happiness, surprise, fear, disgust, anger, sadness, pain, and pleasure (“expression”). The faces were crossed with the four factors. The rating scale was a linear scale with keywords at each end, serving as axes for the rating scale (i.e. young-old, male-female, neutral-strongest imaginable for each age, sex, expression block of trials).

#### Video-based multiattribute social judgement task "task-shortvideo."

##### Task procedures

The video-based multiattribute social judgement task is a theory of mind task in which participants are prompted to make three different types of assessments about a featured character from a video clip: 1) similarity, 2) likeability, 3) mental state attribution. Participants were familiar with the featured character because the short video clips were pulled from full-length videos that participants had watched in previous sessions, specifically during task-alignvideo. Participants rated three types of ratings per video: 1) perceived similarity towards the character (“similarity”), worded as “How similar are you to this character?”; 2) likeability towards the character (“likeability”) worded as “How much do you like this character?”; and 3) inferring what the character is thinking based on a question prompt, such as “Did the character feel in danger?” or “Was the character remembering something?” (“mental state attribution”). At the beginning of each block, participants were visually presented with one of these three questions prior to watching a set of three character-videos. Subsequently, participants were given a rating cue (e.g. “how similar?”) and were shown a five-second video clip of the character. Once the clip ended, participants had five-seconds to respond to the question using a modified version of the semi-circular gLMS rating scale. To ensure participants were clear about which character the experiment referred to, several measures were implemented: 1) The characters were introduced in videos during earlier sessions, 2) participants rewatched these videos during a pre-scan instructional session, and 3) a slideshow was used to highlight the face of the character being discussed, after participants completed rewatching the videos. We asked participants recognition of the characters; videos were replayed if the participants were not able to recognize the said character. Repeating video stimuli to support video recognition during subsequent character judgment provides an additional avenue: memory-related processes, specifically linking videos across previous sessions of the Naturalistic video viewing task “task-alignvideo”. In total, each run was designed to last 12 minutes and 23 seconds, i.e., 1616 TRs. The task was administered within a single session, during ses-03.

##### Stimuli and scales

Participants were presented with a series of short video clips, ranging from 4.1 to 6.9 seconds (“video”). As mentioned, these video clips were pulled from previous sessions, and edited to single out a character of interest. Immediately following each video clip, participants were prompted to provide character assessments using a modified version of the gLMS (“rating”). The modified gLMS rating scale included the following anchors: Not at all, barely detectable, weak, moderate, high, very high, strongest possible similarity/likability. For mental ease on rating self-referential thoughts on the videos, we cued participants with the type of rating – likeability, similarity, mental state attribution – at the beginning of a video. Afterwards, three trials consecutively asked the same questions. In other words, a rating question was first presented, followed by one video, then followed by a shorter question cue, and this was iteratively done for three consecutive trials.

#### Cognitive and Theory of mind task set "task-fractional"

The Cognitive and Theory of mind (ToM) task set consisted of four subtasks: attention reorienting^21^ (N = 48), memory encoding/retrieval (N = 52), text-based theory of mind^22^ (N = 51), and image-based theory of mind^23^ (N = 47). The purpose of this task was to select and compare across subtasks that are known to engage the angular gyrus. Each participant was pseudo-randomly assigned to undergo two subtasks, which were counterbalanced across participants. In other words, one set of participants completed the attention reorienting task and image-based theory of mind task, while a different participant completed a text-based theory of mind task and memory/encoding task. This task was completed within one session, during ses-04.

**Cognitive/ToM task A: Attention reorienting task** 'runtype-posner'

##### Task procedures

This subtask was a canonical spatial cueing paradigm^21^, which consists of three phases: fixation phase, cue phase, and target search phase. The goal is to identify and indicate the location of the *target* as quickly as possible. A cue precedes the target, designed to highlight the location of the upcoming target. A valid cue correctly precedes the location of the target, facilitating the detection of a target; conversely, an invalid cue incorrectly signals the location of the target. To elaborate on each phase of a given trial, during the fixation phase, participants were presented with a fixation cross and two white-outlined empty square boxes, positioned in the visual fields on the left and right. This was displayed for a jittered duration, averaging around 2.5 seconds (“fixation”). During the cue phase, one of the boxes was highlighted with a green color for 0.2 seconds (“cue”). During the target search phase, a green circle, the target, appeared in the center of the square box on the left or right for 2 seconds (“target”). If the participant responded within this 2-second window, a feedback screen highlighted the box they selected for 0.5 seconds. Afterwards, the next trial begins, with the presentation of the fixation cross and empty white boxes on the screen. A total of 120 trials were performed on this task. On average, the proportion of valid to invalid cues was equal, with a 50/50 distribution across participants (M = 50.82, SD = 5.55). The sequence of valid and invalid cues was generated using a random walk procedure, ensuring variation across participants. This approach was chosen to align with the structure of the task-social paradigm, where high and low cues also occur with equal probability. This design deviates from the original Posner-cueing paradigm, which typically includes 80% valid cues^21^. This approach was chosen to align with the structure of the task-social paradigm, where high and low cues also occur with equal probability. This aligns with the overarching aim of the task: to examine how participants dynamically adjust their attention based on changing cue informativeness, rather than relying on consistent probabilistic structure. In total, each run was designed to last 10 minutes and 8 seconds, i.e., 1322 TRs.

##### Stimuli and scales

Stimuli were simple geometric shapes, i.e. boxes and circles. In this subtask, no rating scale was used. Participants responded to the target using the two buttons of an MR-compatible trackball. Code was custom-developed for this task.

**Cognitive/ToM task B: Memory encoding-retrieval task** 'runtype-memory'

##### Task procedures

This subtask was designed to be identical to encoding retrieval tasks, which consist of three phases, repeated twice: a memory encoding phase, a memory distraction phase, and a memory recall phase. The memory encoding phase entailed the presentation of a small illustration clip-art presented for 1 second each (“encoding”), with a total of 26 × 2, i.e., 52 images. The memory distraction phase, intended to prohibit active memory maintenance, included a math calculation task with two math problems × 2 (“distraction”), each displayed for 25 seconds followed by a 5 second response epoch. During the memory recall phase, participants were presented with clip-arts one by one, with a total of 40 × 2 images, and were asked whether they had seen each clip-art before or not. Participants then responded “old” or “new” by pressing the appropriate button under each clip-art. Each response phase lasted 2 seconds (“retrieval”). In total, each run was designed to last 10 minutes and 8 seconds, i.e., 1322 TRs. Due to the recency and primary memory effects, the first and last three images in the encoding test are labeled as null trials.

##### Stimuli and scales

Stimuli were image-based drawings of everyday objects or animate beings. No rating scale was used. Participants responded to the target using the two buttons of an MR-compatible trackball, indicating “old” or “new” to the image presented on screen. Code was custom-developed for this task.

**Cognitive/ToM task C: Image-based theory of mind task** 'runtype-tomspunt'

##### Task procedures

This subtask was derived from Spunt & Adolphs’ theory of mind why/how localizer^23^ to investigate mental state attribution. The experiment focuses on two cognitive aspects of observable actions: mentalizing the implicit mental states driving the actions (“why”) and describing the explicit physical attributes of these actions (“how”). In each trial, participants first saw a prompt (“question”) for 2.1 seconds, followed by a photo (“photo”) with a duration of 1.75 seconds, and then answered yes/no to the prompted question. In total, each run was designed to have 256 images, with 64 images crossed with 2 questions (why/how) and 2 mediums (face/hand), and lasted 10 minutes and 8 seconds, i.e., 1322 TRs.

##### Stimuli and scales

An example of a stimulus includes a photo that depicts an individual looking sideways. One question would ask “Is this person looking away? (how)” and another would ask “Is this person expressing doubt? (why)” The same image was yoked with the 2 (why/how) × 2 (face/hand) combination of questions, which engaged different processes related to physical vs. mental state attribution. Stimuli, scales, and code are available via 10.5281/zenodo.50244.

**Cognitive/ToM task D: Text-based theory of mind task** 'runtype-tomsaxe'

##### Task procedures

This subtask was derived from Dodell-Feder, Dufour, and Saxe’s theory of mind false belief localizer task^22^ to investigate theory of mind processes, i.e., the process of representing and attributing mental states of another agent. Participants read anecdotes of false beliefs and false photographs and answered a true/false questions regarding the stories. In each trial, a fixation cross was presented for 12 seconds (“fixation”), after which participants read an anecdote for 14 seconds (“story”) and responded to a question regarding the anecdote, presented on screen for 10 seconds (“question”). In total, each run was designed to last 10 minutes and 8 seconds, i.e., 1322 TRs.

##### Stimuli and scales

“False belief” stories were narratives where the reader had to represent outdated beliefs of an agent’s latent thought to achieve a correct understanding. For example, “Tom usually takes a left turn on the main road to get to work. Little did Tom know that, this morning, the left section of the main road was under construction.” “False photo” stories involved interpreting false or outdated content in a photograph. For example, an old photograph depicts the photo of an island, but no longer accurately describes the current state due to a volcanic erruption. “Questions” were structured as true/false statements; “true/false” keywords were displayed on-screen. For a statement like “Tom makes a right turn on the main road to avoid the construction”, or ”The island still exists in its beautiful form” participants responded by pressing a button to indicate their answer, either true or false. Stimuli, scales, and code are available via https://saxelab.mit.edu/use-our-efficient-false-belief-localizer/.

### MRI data acquisition

All fMRI data were acquired on a 3T Siemens MAGNETOM Prisma MRI scanner with 32-channel parallel imaging at the Dartmouth Brain Imaging Center at Dartmouth College. Structural images were acquired using high-resolution T1 spoiled gradient recall images and were used for anatomical localization and warping to the standard Montreal Neurological Institute (MNI) space only. Functional images were acquired with a multiband EPI sequence (repetition time = 460 ms, echo time = 27.2 ms, field of view = 220 mm, multiband acceleration factor = 8, flip angle = 44^°^, 64 × 64 matrix, 2.7 × 2.7 × 2.7 mm voxels, 56 interleaved ascending slices, phase encoding posterior  >> anterior). Stimulus presentation and behavioral data acquisition were controlled using Psychtoolbox (MATLAB, MathWorks). Magnetic resonance imaging acquisition parameters are listed in Table 2.

### MRI data curation

MRI protocols in the scanner were renamed to follow the ReproIn naming convention, ensuring robust and automated conversion into BIDS format using HeuDiConv v.0.9.0^24^. For initial sequences not named according to ReproIn, we provided a remapping to facilitate conversion. All participant IDs were anonymized to follow within study sequential order from 1 to 133, left padded with 0 to the length of four digits, e.g. sub-0123. Data was converted to BIDS DataLad dataset using HeuDiConv with dcm2niix v.1.0.20201102^25^, shipped within Singularity container of the ReproNim/containers DataLad dataset^26^ repronim-reproin–0.9.0.sing.

### MRI preprocessing

Preprocessing was performed using fMRIPrep 21.0.2^27^ which is based on Nipype 1.6.1^28^,^29^. The following description in this section is generated from the standard fMRIPrep preprocessing boilerplate text to ensure a consistent description across different processing pipelines.

#### Preprocessing of B0 inhomogeneity mappings

A *B0*-nonuniformity map (or *fieldmap*) was estimated based on two (or more) echo-planar imaging (EPI) references with topup [FSL 6.0.5.1: 57b01774]^30^.

#### Anatomical data preprocessing

A total of 1 T1-weighted (T1w) images were found within the input BIDS dataset.The T1-weighted (T1w) image was corrected for intensity non-uniformity (INU) with N4BiasFieldCorrection^31^, distributed with ANTs 2.3.3 [RRID: SCR_004757]^32^, and used as T1w-reference throughout the workflow. The T1w-reference was then skull-stripped with a *Nipype* implementation of the antsBrainExtraction.sh workflow (from ANTs), using OASIS30ANTs as target template. Brain tissue segmentation of cerebrospinal fluid (CSF), white-matter (WM) and gray-matter (GM) was performed on the brain-extracted T1w using fast [FSL 6.0.5.1: 57b01774, RRID: SCR_002823]^33^. Brain surfaces were reconstructed using recon-all [FreeSurfer 6.0.1,RRID: SCR_001847]^34^, and the brain mask estimated previously was refined with a custom variation of the method to reconcile ANTs-derived and FreeSurfer-derived segmentations of the cortical gray-matter of Mindboggle [RRID: SCR_002438]^35^. Volume-based spatial normalization to two standard spaces (MNI152NLin2009cAsym, MNI152NLin2009cAsym) was performed through nonlinear registration with antsRegistration (ANTs 2.3.3), using brain-extracted versions of both T1w reference and the T1w template. The following templates were selected for spatial normalization: *ICBM 152 Nonlinear Asymmetrical template version 2009c* [^36^, RRID: SCR_008796; TemplateFlow ID: MNI152NLin2009cAsym], *FSL’s MNI ICBM 152 non-linear 6th Generation Asymmetric Average Brain Stereotaxic Registration Model*^37^ mni152nlin6asym, RRID: SCR_002823; TemplateFlow ID: MNI152NLin6Asym].

#### Functional data preprocessing

For each of the 41 BOLD runs found per participant (across all tasks and sessions), the following preprocessing was performed. First, a reference volume and its skull-stripped version were generated by aligning and averaging 1 single-band references (SBRefs). Head-motion parameters with respect to the BOLD reference (transformation matrices, and six corresponding rotation and translation parameters) are estimated before any spatiotemporal filtering using mcflirt [FSL 6.0.5.1: 57b01774]^38^. The estimated *fieldmap* was then aligned with rigid-registration to the target EPI (echo-planar imaging) reference run. The field coefficients were mapped on to the reference EPI using the transform. The BOLD reference was then co-registered to the T1w reference using bbregister (FreeSurfer) which implements boundary-based registration^39^. Co-registration was configured with nine degrees of freedom to account for distortions remaining in the BOLD reference. First, a reference volume and its skull-stripped version were generated using a custom methodology of *fMRIPrep*. Several confounding time-series were calculated based on the *preprocessed BOLD*: framewise displacement (FD), DVARS and three region-wise global signals. FD was computed using two formulations following Power (absolute sum of relative motions^40^) and Jenkinson (relative root mean square displacement between affines^38^). FD and DVARS are calculated for each functional run, both using their implementations in *Nipype* following the definitions by Powers and colleagues^40^. The three global signals are extracted within the CSF, the WM, and the whole-brain masks. Additionally, a set of physiological regressors were extracted to allow for component-based noise correction *CompCor*^41^. Principal components are estimated after high-pass filtering the *preprocessed BOLD* time-series (using a discrete cosine filter with 128s cut-off) for the two *CompCor* variants: temporal (tCompCor) and anatomical (aCompCor). tCompCor components are then calculated from the top 2% variable voxels within the brain mask. For aCompCor, three probabilistic masks (CSF, WM and combined CSF+WM) are generated in anatomical space. The implementation differs from that of Behzadi *et al*. in that instead of eroding the masks by 2 pixels on BOLD space, the aCompCor masks are subtracted a mask of pixels that likely contain a volume fraction of GM. This mask is obtained by dilating a GM mask extracted from the FreeSurfer’s *aseg* segmentation, and it ensures components are not extracted from voxels containing a minimal fraction of GM. Finally, these masks are resampled into BOLD space and binarized by thresholding at 0.99 (as in the original implementation). Components are also calculated separately within the WM and CSF masks. For each CompCor decomposition, the *k* components with the largest singular values are retained, such that the retained components” time series are sufficient to explain 50 percent of variance across the nuisance mask (CSF, WM, combined, or temporal). The remaining components are dropped from consideration. The head-motion estimates calculated in the correction step were also placed within the corresponding confounds file. The confound time series derived from head motion estimates and global signals were expanded with the inclusion of temporal derivatives and quadratic terms for each^42^. Frames that exceeded a threshold of 0.9 mm FD or 1.5 standardised DVARS were annotated as motion outliers. The BOLD time-series were resampled into standard space, generating a *preprocessed BOLD run in MNI152NLin2009cAsym space*. First, a reference volume and its skull-stripped version were generated using a custom methodology of *fMRIPrep*. The BOLD time-series were resampled onto the following surfaces (FreeSurfer reconstruction nomenclature): *fsaverage*. *Grayordinates* files^43^ containing 91k samples were also generated using the highest-resolution fsaverage as intermediate standardized surface space. All resamplings can be performed with *a single interpolation step* by composing all the pertinent transformations (i.e. head-motion transform matrices, susceptibility distortion correction when available, and co-registrations to anatomical and output spaces). Gridded (volumetric) resamplings were performed using antsApplyTransforms (ANTs), configured with Lanczos interpolation to minimize the smoothing effects of other kernels^44^. Non-gridded (surface) resamplings were performed using mri_vol2surf (FreeSurfer).

### Pre-session questionnaires

To connect neuroimaging and behavioral parameters to general psychosocial characteristics, we administered a battery of questionnaires prior to participants’ first visit (Table 3).

### Task instructions

During the behavioral instruction phase, we ensured that participant were informed of the same instructions, despite being introduced to different individuals. Therefore, scripted dialogues were provided to experimenters to be verbally read aloud (hosted on: https://github.com/spatialtopology/calibrate/tree/main/dialogue). This was accompanied by visual aids presented on a monitor in front of participants. Afterwards, participants would complete practice tasks, which are located in github repositories (https://github.com/spatialtopology/task-alignvideo/blob/main/scripts/RUN_alignvideos.m; https://github.com/spatialtopology/fractional_factorials/blob/master/RUN_practice.m).

#### Scan setup

##### Audio Calibration

To ensure that auditory stimuli delivered during the experimental tasks would be presented at a volume that was perceptible to the participant, prior to each scan session, participants completed a brief audio calibration task. During initial structural scans (i.e., scout, PEpolar scans), a video clip was played and the participant provided a rating to indicate whether the volume was set to a satisfactory level (https://github.com/spatialtopology/calibrate/blob/main/scripts/RUN_audio_calibrate.m). After the audio calibration task was complete, the experimenters verbally confirmed with the participant that the final volume level was adequate.

##### Arm Measurement Procedures for Application of Thermal Device

Two arm sites were identified for the administration of thermal stimuli during the multimodal negative affect task-social task. To ensure the same sites were used reliably across sessions, we developed an arm measurement protocol. First, the experimenter took an initial measurement along the midline of the participants left arm: from the crease of the elbow to the crease of the wrist. Using this baseline measurement, the experimenter marked two primary sites, located 1/3 and 2/3 of the distance from the elbow to the wrist. The thermode was applied to these sites and the participant completed a pain screening procedure to verify that they could tolerate the noxious stimuli used during the study. If participants reported hypersensitivity or hyposensitivity on either of the primary arm sites, a secondary site was used, 3 cm below the primary site locations. If hypersensitivity or hyposensitivity was also reported at the secondary arm sites, then the participant was removed from the experiment.

### Preprocessing computing environment

The BIDS-formatted data was analyzed at the Johns Hopkins University Joint High Performance Computing Exchange, a high-performance cluster running CentOS Linux 7.9 and the Sun Grid Engine scheduler. On the Exchange, software packages are made accessible via lmod^45^. For this dataset, job submissions were managed with targets^46^, which interfaces with the scheduler via batchtools^47^. These packages were tracked via renv^48^. Each job consisted of a call to a Singularity image^49,50^ which implemented one of the analyses described above (e.g., fMRIPrep, MRIQC). The derivatives of these apps were tracked by git-annex and DataLad^51^.

## Data Records

The unprocessed source data — with corresponding metadata, events files, and stimuli — are publicly available on OpenNeuro (https://openneuro.org/datasets/ds005256/)^52^. The dataset is organized abiding the Brain Imaging Data Structure (BIDS)^53^ version 1.9.0, which allows for standardized file naming convention and storage structure for accessibility and reproducibility across neuroimaging datasets. Further details on the BIDS format can be found at https://bids-specification.readthedocs.io/en/v1.9.0/. Participants are indexed with numerical identifiers (e.g. sub-0001) and scanned across multiple sessions (ses-01, ses-02, etc). Within each session, different tasks were performed (task-alignvideos, task-narratives, task-faces, etc.), with multiple runs for each task (run-01, run-02 etc.). Each run may vary in task instructions or counterbalancing schemes depending on the task- type (see subsection “Specific Tasks” for further details).

At the participant level, session-specific subdirectories (ses-01 to ses-04) contain folders for anatomical (anat), (func), and (fmap) data. T1-weighted images and diffusion weighted images are stored under anat/ (collected only during ses-01), functional EPI images are under func/ (available for all sessions), and corresponding B0 maps are hosted under fmap/, which can be utilized for further distortion correction. Sidecar JSON files accompanying these images provide metadata on MRI acquisition parameters. For each functional data, _events.tsv files document timestamps and metadata for trial event during functional tasks. Each session directory also includes a _scans.tsv file and corresponding JSON file, which documents filename and task conditions that were operated on a run- level. (For example, the column task-social_runtype within _scans.tsv specifics the subtask out of three subtasks performed within task-social). The top-level directory includes a data_description.json file with metadata on licensing, acknowledgements, and funding. A code/ directory contains scripts used for dataset duration, (available on github at github.com/spatialtopology/spacetop-prep), and a stimuli/ directory hosts all the stimuli presented in the functional tasks, organized by task type. A README and CHANGE file provide additional dataset documentation.

This dataset was curated using DataLad (https://www.datalad.org/), whih integrates git and git-annex on large scale datasets. DataLad allows users to keep track of any changes made to the source data files and its derivatives while enabling subdatasets and its independent download, manipulation or purging of subdatasets, such as code/ and stimuli/.

## Technical Validation

### Preprocessing metrics

**Fig. 5:**
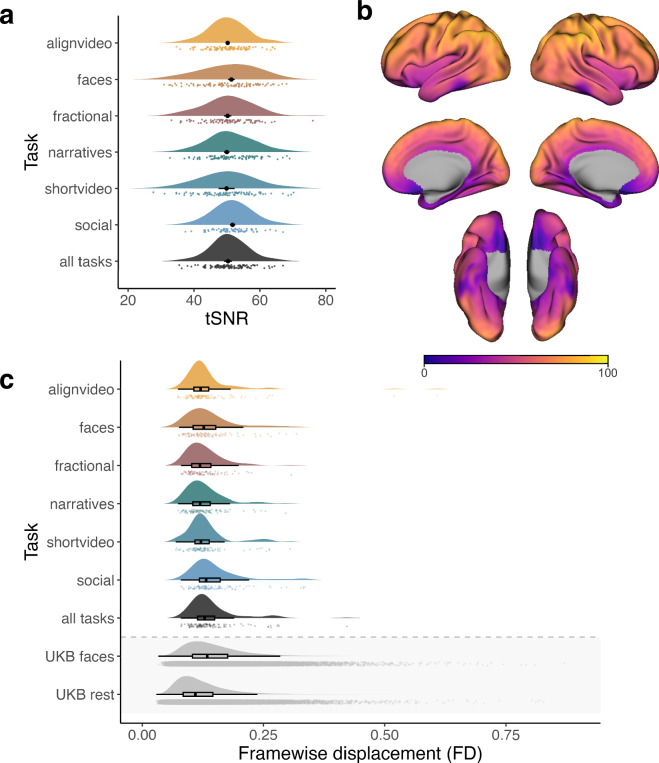
Image quality metrics. **(a)** Temporal signal to noise ratio (tSNR) was calculated within-subject runs and aggregated across participants. Each density plot represents the distribution of tSNR median values across participants, also reflected in the task-corresponding color markers. The black color markers indicate the group average median tSNR value, calculated across within-mask voxels. The black error bars represent the within-subjects confidence interval for the median tSNR value; Given that task-shortvideo has one run in the experiment, the error bar reflects the between-subject confidence interval. As indicated in the plot, median tSNR confidence intervals shrink as a function of repeated runs (alignvideos 13 runs; faces 3 runs; fractional 2 runs; narratives 4 runs; shortvideo 1 run; social 18 runs; all tasks 41 runs). **(b)** tSNR of align video task across the cortical surface. Colors indicate median tSNR per voxel. tSNR maps were computed using the preprocessed MNI runwise maps. Groupwise median tSNR maps were converted to fsaverage6 space, using neuromaps, for visualization. **(c)** Head movement is kept to a minimum, reflected in framewise-displacement (FD) values. Each density plot represents the distribution of FD values across participants, also reflected in the task-corresponding color markers. The box plot represents the median value and interquartile range of the group level FD distribution. UKbiobank (UKB) FD values are displayed here for comparison; Across all tasks, the current dataset FD values are lower and less variable compared to that of the “UKB faces” task. “UKB rest” condition has a slightly lower median as it serves as a no task condition compared to the tasks in our current dataset. Overall, head movement was kept to a minimum, as demonstrated by framewise displacement lower than that of the UK biobank, in spite of the longer scan protocol.

For quality control, we used MRIQC BIDS-App^54^ and extracted image quality metrics (IQMs). To demonstrate quality of the data, we focus on temporal signal to noise ratio (tSNR) and framewise displacement (FD). For the tSNR, per run, we calculate the mean image and temporal standard deviation image and divide the two. The mean image was generated from fMRIPrep, the standard deviation map was generated using fslmaths. These tSNR maps are aggregated to the participant level (Fig. 5a). The tSNR maps are fairly homogenous across tasks, so we have plotted the median tSNR map from the task-alignvideo as a reference (Fig. 5b). In addition, we extract the framewise displacement values per run, average within-participant, and aggregate it to the group level (Fig. 5c). Each task’s median FD value is smaller than 0.2 mm, and comparable or smaller than that when compared to the FD values of a large scale dataset, UKbiobank^55^.

### Statistical maps

**Fig. 6:**
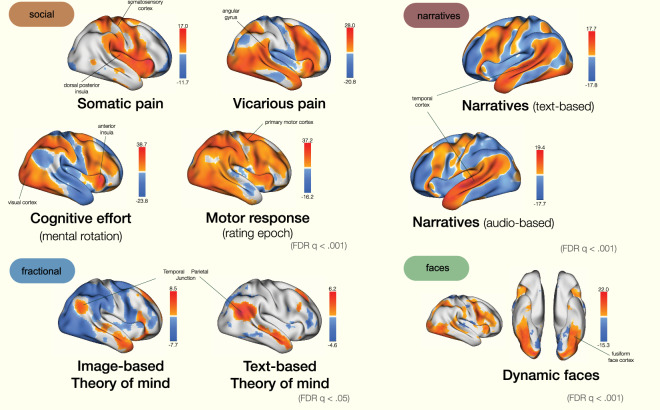
Statistical maps of canonical contrasts across four tasks. Group-average statistical maps from each task suggests robust activation that aligns with past literature/activation patterns. Each map is constructed by modeling conditions of interest as a box car function (e.g. somatic pain, vicarious pain, faces), group-averaged across participants, and thresholded at FDR q < 0.001 or q < 0.05. Within task-social, we see canonical activation in the somatosensory and dorsal posterior insula for somatic pain conditions. Within Task-fractional, the temporal cortex displays robust activation. task-social statistical maps involve activation in the temporal parietal junction across text- and image-based theory of mind tasks. Task-faces recruits the fusiform face cortex.

For data quality validation, we analyzed general linear model (GLM) contrast maps from four different tasks (Fig. 6). We primarily aimed to evaluate that the statistical maps showed robust effects consistent with prior research. Each statistical map is a group-level average of specific contrasts, in which the condition of interest was modeled as a boxcar function, contrasted against an implicit baseline of fixation epochs, and group-averaged across subjects, later thresholded (FDR *q* < 0.001 for task-social, task-narratives, and task-faces as the sample size includes the entire dataset; FDR *q* < 0.05 for task-fractional as the sample size includes a subset of the dataset) For task-social, we present conditions of somatic pain, vicarious pain, and cognitive effort during stimulus presentation, contrasted against an implicit baseline. The motor response is when the trackball button was pressed. In the narratives task, we estimated the neural correlates of reading and listening to the narratives, resulting in robust activation in the temporal cortices. The task-faces statistical map recruits greater activation in the fusiform cortices when participants view faces compared to an implicit baseline; lastly, robust activation in the temporal parietal junction and prefrontal cortices are notable during task-fraction for both image-based and text-based theory of mind tasks.

## Usage Notes

This dataset is accessible on OpenNeuro using the following link: https://openneuro.org/datasets/ds005256/versions/1.1.0^52^. Users can download the dataset using the web browser or via command line tools. Using openneuro-cli: openneuro download –snapshot 1.0.0 ds005256 ds005256-download/. Further usage details on OpenNeuro datasets can be found at https://docs.openneuro.org/user_guide.html#viewing-and-downloading-a-dataset. Users can also download the dataset via DataLad: datalad installhttps://github.com/OpenNeuroDatasets/ds005256.git.

## Data Availability

Code for stimulus presentation and data acquisition is published in a GitHub repository (https://github.com/spatialtopology). To ensure reproducibility of the experimental procedures, we released the code as version 1, prior to data collection (“task-alignvideo”: https://github.com/spatialtopology/alignvideos/releases/tag/v1.0.0-stable, “task-social”: https://github.com/spatialtopology/social_influence/releases/tag/v1.0.0-stable, “task-faces”: https://github.com/spatialtopology/task-faces/releases/tag/1.0.0, “task-narratives”: https://github.com/spatialtopology/narratives/releases/tag/v.1.0.0, “task-shortvideo”: https://github.com/spatialtopology/shortvideos/releases/tag/v1.0.0-stable, “task-fractional”: https://github.com/spatialtopology/fractional_factorials/releases/tag/v.1.0.0). Code for data wrangling, preprocessing, and analyzing the data is in the GitHub repository (https://github.com/spatialtopology/spacetop-prep). Neuroimaging data and behavioral data are BIDS-formatted in DataLad^56^. Code for data wrangling and analyzing the data is in the github repository (https://github.com/spatialtopology/spacetop-prep). Neuroimaging data and behavioral data are BIDS-formatted in DataLad (https://openneuro.org/datasets/ds005256).
